# Supplementary material for: Vaccine Uptake to Prevent Meningitis and Encephalitis in Shanghai, China
Source: Vaccines (Basel). 2022 Nov 30;10(12):2054. doi: 10.3390/vaccines10122054 (PMC9787460; doi:10.3390/vaccines10122054)
Supplement: Supplementary file 1 [file vaccines-10-02054-s001.zip › vaccines-2016322-supplementary.pdf]

Supplementary Text

Validity and Reliability Measurement of Health Belief Model (HBM)

For the HBM, KMO value was estimated to be 0.884, and the HBM constructs were significantly correlated by Bartlett's test of sphericity ($p < 0.0001$). All the Cronbach's α in the reliability of HBM constructs was almost and above 0.6, which was acceptable (Supplementary Material, Table S2). AVE in three HBM constructs was above 0.5 (Supplementary Material, Table S1), and most external factor loadings were above 0.7 (Supplementary Material, Table S3), suggesting good convergent validity. To further evaluate the discriminant validity, we compared the cross loadings within and between the HBM constructs and subscale ratings, which verified the consistency of different items loaded on their specific structures (Supplementary Material, Table S3). Additionally, multicollinearity was excluded as all VIFs of the HBM constructs were less than 5.

Table S1. Six vaccines that may prevent meningitis/encephalitis in children and their manufacturers.

Vaccines	Types	Manufacturers
Meningococcal polysaccharide vaccine (MPSV) / meningococcal polysaccharide conjugate vaccine (MPCV)	MPSV-A (EPI)	Wuhan Institute of Biological Products Co., Ltd.
	MPSV-AC (EPI)	Lanzhou Institute of Biological Products Co., Ltd.
	MPSV-AC (EPI)	Walvax Biotechnology Co.,Ltd.
	MPCV-AC	Royal (Wuxi) Bio-Pharmaceutical Co.,Ltd.
	MPCV-AC	Chongqing Zhifei Biological Products Co.,ltd.
	MPCV-AC	Walvax Biotechnology Co., Ltd.
	MPCV-AC/Hib	Chongqing Zhifei Biological Products Co.,ltd.
	MPSV-ACY135	Chongqing Zhifei Biological Products Co.,ltd.
	MPSV-ACY135	Chengdu Kanghua Biological Products Co., Ltd.
	MPSV-ACY135	Walvax Biotechnology Co., Ltd.
	MPCV-ACYW135	Cansino Biologics Inc.
Japanese encephalitis vaccine (JEV)	Live attenuated JEV (EPI)	Wuhan Institute of Biological Products Co., Ltd.
	Live attenuated JEV (EPI)	Chengdu Institute of Biological Products Co., Ltd
	Inactivated JEV (Vero cell), freeze-dried	Liaoning Chengda Biotechnology Co., Ltd.
13-valent pneumococcal conjugate vaccine (PCV13)/23-valent pneumococcal polysaccharide vaccine (PPSV23)	PCV13	Pfizer Inc.
	PCV13	Walvax Biotechnology Co., Ltd.
	PPSV23	Sinovac Biotech Co., LTD.
	PPSV23	Chengdu Institute of Biological Products Co., Ltd
	PPSV23	Merck & Co., Inc.
	PPSV23	Walvax Biotechnology Co.,Ltd.
	PPSV23	Beijing Minhai Biotechnology Co., Ltd
Haemophilus influenzae type b (Hib)-containing vaccines	Hib conjugate vaccine	Lanzhou Institute of Biological Products Co., Ltd.
	Hib conjugate vaccine	SANOFI PASTEUR S.A.
	DTP-IPV-Hib vaccine	SANOFI PASTEUR S.A.
	Hib conjugate vaccine	Walvax Biotechnology Co.,Ltd.
	Hib conjugate vaccine, freeze-dried	Beijing Minhai Biotechnology Co., Ltd
	DTP-Hib vaccine	Beijing Minhai Biotechnology Co., Ltd

Vaccines	Types	Manufacturers
	Hib conjugate vaccine	Beijing Zhifei lvzhu Biopharmaceutical Co.,Ltd.
	MPCV-AC/Hib	Beijing Zhifei lvzhu Biopharmaceutical Co.,Ltd.
	Hib conjugate vaccine	Chengdu Olymvax Biopharmaceuticals Inc.
Oral live attenuated rotavirus vaccine	Chinese lamb rotavirus vaccine	Lanzhou Institute of Biological Products Co., Ltd.
	Pentavalent rotavirus vaccine	Merck & Co., Inc.
Inactivated enterovirus 71 (EV-71) vaccine	EV-71 vaccine (Vero cell)	Sinovac Biotech Co., LTD.
	EV-71 vaccine (Vero cell)	Wuhan Institute of Biological Products Co., Ltd.
	EV-71 vaccine (Human Diploid cell)	Institute of Medical Biology, Chinese Academy of Medical Sciences

EPI, expanded program of immunization; DTP, diphtheria-tetanus-pertussis vaccine; IPV, inactivated polio vaccine.
Meningococcal vaccines and Japanese encephalitis vaccines that are included in EPI were labeled with (EPI), and the rest of these two vaccines are non-EPI surrogate vaccines. The other four vaccines are all non-EPI non-surrogate vaccines.

Table S2. Reliability of health belief model (HBM) constructs.

HBM Constructs	Subscale Ratings	Loadings	Cronbach's α	Composite Reliability	Average Variance Extracted (AVE)
Perceived susceptibility	1	0.600	0.583	0.47	0.31
	2	0.506			
Perceived severity	3	0.843	0.899	0.84	0.72
	4	0.855			
Perceived benefits	5	0.510	0.777	0.28	0.45
	6	0.562			
Self-efficacy	7	0.570	0.837	0.70	0.45
	8	0.751			
	9	0.670			
Perceived barriers	10	0.582	0.791	0.77	0.54
	11	0.891			
	12	0.700			
Cues to action	13	0.812	0.866	0.79	0.65
	14	0.805			
	15	0.701	0.882	0.68	0.52
	16	0.738			

Table S3. External factor loadings of health belief model (HBM) constructs.

Subscale Ratings	Perceived Susceptibility	Perceived Severity	Perceived Benefits	Self-Efficacy	Perceived Barriers	Cues to Action 1 *	Cues to Action 2 *	Vaccine Uptake
**1**	0.506	0.319	0.078	0.303	−0.089	0.218	−0.099	0.191
**2**	0.600	0.024	0.071	0.197	−0.296	0.056	−0.153	−0.058
**3**	0.090	0.843	0.076	0.169	−0.033	0.210	−0.089	0.036
**4**	0.055	0.855	0.049	0.209	−0.017	0.209	−0.088	−0.057
**5**	0.110	0.562	0.435	0.290	−0.025	0.333	−0.109	0.207
**6**	0.190	0.365	0.510	0.310	−0.093	0.347	−0.088	−0.060
**7**	0.124	0.405	0.152	0.570	−0.066	0.395	−0.049	0.040
**8**	0.139	0.161	0.133	0.751	−0.207	0.209	−0.078	−0.001
**9**	0.101	0.246	0.061	0.670	−0.157	0.229	−0.089	0.156
**10**	0.012	−0.161	0.004	−0.186	0.582	−0.142	0.209	−0.130
**11**	−0.135	−0.004	−0.075	−0.016	0.891	−0.021	0.169	−0.054
**12**	−0.135	0.052	0.005	−0.025	0.700	−0.018	0.218	0.120
**13**	−0.100	−0.111	−0.048	−0.061	0.255	−0.117	0.812	0.027
**14**	−0.079	−0.087	−0.025	−0.027	0.322	−0.080	0.805	−0.051
**15**	0.118	0.328	0.093	0.307	−0.124	0.738	−0.141	−0.021
**16**	0.098	0.374	0.137	0.325	−0.056	0.701	−0.138	0.046
**Vaccine uptake**	0.160	0.047	0.001	0.253	0.086	0.038	0.034	1.000

* In the HBM, grouping of C13 and C14 (cues to action 1), and C15 and C16 (cues to action 2) led to a better discriminant validity.

Survey Questionnaire

A0. Do you have any children aged 1-6 years?

1. No (survey is over, thank you for your time)
2. Yes (please continue)

A. Demographics

A1. Your relationship with your child?

1. Father 2. Mother 3. Grandfather 4. Grandmother

A2. What is your birth year? ______

A3. Where is your registered residence?

______province______city/prefecture/region

A4. What is your ethnic group?

A5. What is your highest educational level?

1. Primary school 2. Middle school 3. High school/technical secondary school/technical school/secondary vocational technical school 4. College 5. University (bachelor' degree) 6. Master's degree or above

A6. What is your household (family) monthly income (CNY)?

1. <5000 2. 5000-9999 3. 10000-14999 4. 15000-19999 5. 20000-29999 6. 30000-39999 7. 40000-49999 8. >50000 9. No disclosure

A7. How many children do you have? (If you are a grandparent, how many grandchildren do you have living or caring for together?)

1. One (What is your child's birth year______)
2. Two (What is your first child's birth year______
   What is your second child's birth year______)
3. Three (What is your first child's birth year______
   What is your second child's birth year______
   What is your third child's birth year______)

B. Diseases and vaccines

B1. Has your child ever suffered from the following diseases?

B11. Meningitis or encephalitis 1. Yes 2. No 3. I don't know

B12. Pneumonia 1. Yes 2. No 3. I don't know

B13. Diarrhea 1. Yes 2. No 3. I don't know

B14. Hand-foot-and-mouth disease 1. Yes 2. No 3. I don't know

B2. What are the common manifestations of meningitis/encephalitis in children? (There may be more than one correct answers)

1. Headache, dizziness, fever, nausea, vomiting
2. Mental symptoms, personality change, memory impairment, abnormal physical activity, irritability, convulsions, respiratory disorders, incontinence
3. There may be no obvious symptom
4. I don't know

B3. Which of the following pathogens may cause meningitis/encephalitis in children? (There may be more than one correct answers)

1. Meningococcus
2. Japanese encephalitis virus
3. Streptococcus pneumonia
4. *Haemophilus influenzae* type b
5. Rotavirus
6. Enterovirus-71
7. Herpes simplex virus
8. Varicella zoster virus
9. Measles virus
10. Mumps virus

B4. What is the possible transmission route of the following pathogens? (There may be more than one correct answers)

	Airborne	Food-borne	Close contact	Mosquito-borne	Blood-borne
Meningococcus					
Japanese encephalitis virus					
Streptococcus pneumonia					
Haemophilus influenzae type b					
Rotavirus					
Enterovirus-71					
Herpes simplex virus					
Varicella zoster virus					
Measles virus					
Mumps virus					

B5. What diseases could be prevented by the following vaccines? (There may be more than one correct answers)

	Meningitis/ Encephalitis	Pneumonia	Diarrhea	Hand-foot-and-mouth disease
Meningococcal vaccine (Epidemic M/E vaccine in Chinese)				
Japanese encephalitis vaccine (Category II M/E vaccine in Chinese)				
Pneumococcal vaccine (13-valent/23-valent)				
Hib-containing vaccine (Hib vaccine, quadruple vaccine or pentavaccine)				
Rotavirus vaccine				
EV-71 vaccine				

C. Health beliefs

Vaccines that may prevent M/E (M/E vaccines) include meningococcal vaccine (epidemic M/E vaccine in Chinese), Japanese encephalitis vaccine (category II M/E vaccine in Chinese), pneumococcal vaccine (13-valent/23-valent), Hib-containing vaccine (Hib vaccine, quadruple vaccine or pentavaccine), rotavirus vaccine, and EV-71 vaccine.

		Strongly agree	agree	neutral	disagree	Strongly disagree
C1	Risk of getting M/E is very high for children.					
C2	My child is very susceptible to getting M/E.					
C3	M/E has a very serious impact on children's health.					
C4	Children feel very sick when they get M/E.					
C5	M/E vaccines effectively protect children from infection.					
C6	M/E vaccines are beneficial for children's life and study.					
C7	My child can receive M/E vaccines.					
C8	Chance of adverse reactions to M/E vaccines is low.					
C9	Physicians may properly handle potential adverse reactions to M/E vaccines.					
C10	M/E vaccination is expensive.					
C11	M/E vaccination service is inconvenient.					
C12	M/E vaccines may have long-term adverse effects in children.					
C13	I have no idea of pathogens that may cause M/E in children.					
C14	I have no idea of vaccines that may prevent M/E in children.					
C15	I am likely to vaccinate my child when more information is available on M/E and vaccines.					
C16	I am likely to vaccinate my child when M/E vaccines are more widely accepted.					

D. Vaccine uptake

Has your child received any of the following vaccines?

D11. Meningococcal vaccine

1. Yes 2. No (if your answer is "yes", please response to D21)

D21. Which type of meningococcal vaccine has your child received?

1. Free vaccine
2. Self-paid vaccine
3. I don't know

D12. Japanese encephalitis vaccine

1. Yes 2. No (if your answer is "yes", please response to D22)

D22. Which type of Japanese encephalitis vaccine has your child received?

1. Free vaccine
2. Self-paid vaccine
3. I don't know

D13. Pneumococcal vaccine (13-valent/23-valent)

1. Yes 2. No (if your answer is "yes", please response to D23)

D23. Which type of Pneumococcal vaccine has your child received?

1. 13-valent
2. 23-valent
3. I don't know

D14. Hib-containing vaccine (Hib vaccine, Quadruple vaccine or Pentavaccine)

1. Yes 2. No (if your answer is "yes", please response to D24)

D24. Which type of Hib containing vaccine has your child received?

1. Hib vaccine
2. Quadruple
3. Pentavaccine
4. I don't know

D15. Rotavirus vaccine

1. Yes 2. No (if your answer is "yes", please response D25)

D25. Which type of Hib containing vaccine has your child received?

1. Domestic vaccine
2. Imported vaccine
3. I don't know

D16. EV-71 vaccine

1. Yes 2. No
